# An artificial gorilla troops optimizer for stochastic unit commitment problem solution incorporating solar, wind, and load uncertainties

**DOI:** 10.1371/journal.pone.0305329

**Published:** 2024-07-10

**Authors:** Mahmoud Rihan, Aml Sayed, Adel Bedair Abdel-Rahman, Mohamed Ebeed, Thamer A. H. Alghamdi, Hossam S. Salama

**Affiliations:** 1 Electrical Engineering Department, Faculty of Engineering, South Valley University, Qena, Egypt; 2 Electronics and Communications Engineering Department, Egypt-Japan University of Science and Technology, Alexandria, Egypt; 3 Electronics and Communications Engineering Department, Faculty of Engineering, South Valley University, Qena, Egypt; 4 Department of Electrical Engineering, Faculty of Engineering, Sohag University, Sohag, Egypt; 5 Wolfson Centre for Magnetics, School of Engineering, Cardiff University, Cardiff, United Kingdom; 6 Electrical Engineering Department, School of Engineering, Al-Baha University, Al-Baha, Saudi Arabia; 7 Department of Electrical Engineering, Faculty of Engineering, Aswan University, Aswan, Egypt; SR University, INDIA

## Abstract

The unit commitment (UC) optimization issue is a vital issue in the operation and management of power systems. In recent years, the significant inroads of renewable energy (RE) resources, especially wind power and solar energy generation systems, into power systems have led to a huge increment in levels of uncertainty in power systems. Consequently, solution the UC is being more complicated. In this work, the UC problem solution is addressed using the Artificial Gorilla Troops Optimizer (GTO) for three cases including solving the UC at deterministic state, solving the UC under uncertainties of system and sources with and without RE sources. The uncertainty modelling of the load and RE sources (wind power and solar energy) are made through representing each uncertain variable with a suitable probability density function (PDF) and then the Monte Carlo Simulation (MCS) method is employed to generate a large number of scenarios then a scenario reduction technique known as backward reduction algorithm (BRA) is applied to establish a meaningful overall interpretation of the results. The results show that the overall cost per day is reduced from 0.2181% to 3.7528% at the deterministic state. In addition to that the overall cost reduction per day is 19.23% with integration of the RE resources. According to the results analysis, the main findings from this work are that the GTO is a powerful optimizer in addressing the deterministic UC problem with better cost and faster convergence curve and that RE resources help greatly in running cost saving. Also uncertainty consideration makes the system more reliable and realistic.

## 1. Introduction

### 1.1 Background

Power system operation and management represent a major field of the studies of power systems. Power systems’ priority is to continuously provide all system users with power of satisfying-quality without placing them under excessive financial strain and to have a balanced operating system. This causes the solution of deterministic and stochastic unit commitment (UC) to be an important orientation for power systems’ researchers. UC problem is a mixed-integer, and nonlinear optimization problem. It is concerned about estimating the optimal operating states for each power plant’s generating units, as well as the individual output power for planned generation to achieve the minimum operating cost [1, 2]. It should be considered to cover the load demand at minimum operating cost [3]. In some cases, it is required to involve nonlinearity to the operating cost. This can be accomplished by considering the valve point loading effects [4]. In most cases, valve point loading effects are considered in the solution of the economic dispatch problems [5, 6] but not in the UC problem. Recently, the energy generation is one of the world’s most critical concerns due to rising the demand from the energy resources and the non-renewability of existing fuels. Renewable energy (RE) can yield many environmental and economic benefits compared to conventional fuel- based generation systems. The RE sources are recognized as clean and cost-effective energy sources, and they play an important role as a power generation system to diminish the need for fossil fuels [7–9]. The solar and wind energy systems, in particular, have made significant inroads into power systems.

The study of the efficient consumption of wind and solar energies becomes increasingly significantly in power systems as the penetration levels of wind and solar energy continue to rise [10]. Due to the unexpected nature and variable behavior of wind and solar energies, estimation of generation schedule of thermal units in the power system becomes a difficult challenge in modern power management. Different forecasting methodologies have been introduced previously to estimate wind and solar power production [11–14], but the generation of wind and solar power cannot be predicted with a high degree of precision due to intermittent nature of these energies. Between the real power output and the predicted value, there is an inescapable random error. So, for reliable system planning the uncertainties of RE resources should be considered [15]. As a result, dealing with the UC becomes a challenging mission in the system operation process [16]. Also, consideration of load demand uncertainty, as load isn’t constant during the study period, makes it more complicated to adjust the UC to achieve the best timetable. In optimal planning with integrated RE resources, additional constraints should be considered including the down spinning reserve and the adequate ramping capacity constraints to compensate variations in powers of the RE sources.

### 1.2 Comprehensive literature review

Over years, researchers have turned their efforts to employ different optimization approaches (traditional and modern) to solve the UC problem under deterministic and stochastic cases [17–19]. In [20], authors employed the genetic algorithm (GA) using mutation and crossover operators to solve the deterministic unit commitment (DUC). An improved version of lagrangian relaxation algorithm was applied to handle the DUC under various test systems. In [21], extended priority list technique was applied for DUC problem solution through two steps. In [22], the authors introduce an improved Lagrangian relaxation with a new decision maker. The algorithm was applied on the DUC under systems up to 100 units system. The authors of [23] introduced a Greedy Randomized Adaptive Search Procedure to solve the classical DUC. In [24] the DUC was solved by the application of integer-coded GA with test systems up to 100 generating units. In [25], authors applied an improved SA algorithm combined with dynamic economic dispatch to tackle the DUC. SA was applied for the determination of on/off status of the units. In [26], authors introduced hybrid algorithm called hybrid ant system/ priority list technique to tackle the DUC unit commitment problem with consideration of the operation constraints. The technique was applied on systems up to 100-unit system. The authors of [27], introduced the shuffled frog leaping algorithm (SFLA) to solve the DUC to decrease the energy dispatch cost while satisfying the constraints. The authors of [28] introduced a solution for the DUC using hybrid technique combined between particle swarm optimization and grey wolf optimizer under three different systems. In [29], the authors developed an adaptive lagrangian relaxation to tackle the DUC under 10-unit system with a better way for unit substitute. In [30], a hybrid algorithm combined the evolutionary particle swarm optimization with tabu search was introduced to handle the DUC through two levels. In the first level, the time table for operating process was created then, in the second level, the power dispatch was obtained. In [31], the authors introduced the solution of DUC with line flow constraint by application of GA. In [32], a hybrid technique combined priority list with binary particle swarm optimization was proposed to solve the conventional UC. In [33], an improved ant colony optimization was used to tackle the DUC on basis of maximization the profits of the generating companies. Also, Gravitational search algorithm was introduced to solve the DUC [34]. The authors in [35] studied the stochastic UC under load demand uncertainty using sample average approximation. Authors in [36] solved a large scale DUC using multi-cuts outer approximation method. An improved binary version of differential evolution was introduced in [37] to tackle the small scale and large scale DUC under variety of constraints. In [38], small-sized systems were employed to solve DUC with an improved hybrid algorithm combined particle swarm optimization with dragon fly algorithm. Authors of [39] introduced two versions of binary fish migration optimization to overcome the defects of the original algorithm and applied the developed techniques to the DUC. The authors in [40] introduced a solution of the stochastic unit commitment (SUC) with consideration of load uncertainty. The growth of hybrid power systems which combine RE resources with conventional thermal units makes it more challenging and complicated to solve the UC problem. Uncertainties in RE sources forecasting have a serious impact on the UC solution and pose substantial threats to the power system’s control and operation [41]. As a result, modelling RE power variations becomes essential to solve the UC problem with integrated RE sources. The authors in [42] studied the effect of wind power forecasting uncertainty on the UC solution. The authors of [43] applied an enhanced GSA to solve the UC with wind farm and while considering the fluctuations of wind energy production. They represent the uncertainty using Latin Hypercube Sampling (LHS) and the Cholesky decomposition methods. In [44], UC for a combined system with thermal and hydro units and a big wind farm was solved using a newly developed optimizer called Weight-Improved Crazy Particle Swarm Optimization (WICPSO). The simulation for uncertainty of wind power was made using LHS and the Cholesky decomposition methods. In [45], UC was solved by applying a mixed-integer linear programming with integration of battery energy storage units to make up for the unpredictable characteristics of wind energy production. In [46], authors studied DUC with incorporation of wind and solar energies to investigate their effect on the system economy. The study was made using a modified version of priority list technique. In [47], authors solved the UC problem using LR technique while including uncertainty of RE supplies, generator and transmission line breakdowns. In [48] authors proposed a detailed scheme for integrating uncertainty in distributed power systems with integrated RE resources. Loads, solar and wind power predictions, and generator outages are all studied as causes of uncertainty. They solve the SUC problem using GA. In [49], the UC was handled with the Binary Artificial Sheep Algorithm while considering the variability of RE sources with pumped hydro-energy storage. The uncertainty was represented using LHS and the Cholesky decomposition methods. In [50], the authors employed an intelligent search algorithm to handle the UC with load forecast and wind power uncertainty. In [51], the authors introduced a system contained wind turbines and energy storage system. They solved SUC while considering the uncertainty of wind power. The problem model was reformulated to a single level optimization problem with the use of the strong duality theory as a single-level robust mixed integer linear program. As observed, researchers developed various algorithms and introduced hybrid techniques to tackle DUC and SUC problems. One of the modern optimization algorithms developed in 2021 was Artificial gorilla troops optimizer (GTO) [52]. It is an effective algorithm for high dimensional optimization problems. Recently, GTO was applied to solve various hard optimization problems such as implementing of fractional order PID controller for automatic generation control in interconnected power systems in [53] and controlling the frequency fluctuations in micro grids which come from the wind power uncertainty in [54]. In this work, GTO is employed to handle the DUC and the SUC at RE resources and load demand uncertainties.

### 1.3 Objective, research gaps and contributions of the paper

This paper mainly aims to study the SUC with and without the integration of wind and solar energies to investigate their role in the cost saving and their effect on the performance of the power system without disturbing the problem constraints. Also, the uncertainty of load demand and RE resources is considered in this work for more realistic system operation. Also, it is employed to validate the efficacy of GTO to handle DUC with and without consideration of the valve point loading effect which is rarely applied the UC problem to simulate the nonlinearity of the objective function. The validation is made through results comparison with other well-known techniques and presented works in the literature.

From the previous review survey, it is obvious that huge efforts were presented for solving the UC problem. However, to the best of our knowledge, the research gaps in these works Can be outlined as follow:

- All previous introduced studies on the DUC in the review didn’t account for the nonlinearity of the fuel cost function through application of the valve point loading effect (VPE).
- Reference [35] solved the SUC problem at uncertain load demand without incorporation of RE resources.
- References [42–45, 47, 50, 51] solved the SUC with consideration of wind power uncertainty but there was no integration for solar power units into the system. Although, references [44, 45, 51] introduced energy storage systems to the main system to overcome the unpredictability of the wind power.
- Reference [48] solved the SUC at distributed energy systems with consideration of load uncertainty and RE resources uncertainty (wind and solar power) but it ignored the ramp rate constraints for the generation units.
- Reference [46] solved the UC with wind and solar power integration. However, the uncertainties of system were not considered.
- Reference [49] solved the SUC with wind and solar power integration with the uncertainties of RE resources. However, load uncertainty was not taken into consideration.

Based on the listed research gaps in this area, this paper fills these gaps and the main contributions can be stated as follows:

- Solving the DUC using GTO with and without VPE consideration and validating the results through a comparison with other well-known algorithms.
- Solving the SUC problem with and without inclusion of RE resources.
- Solving the SUC at uncertainties of load demand, wind power, and solar energy.
- Analyzing the system performance with and without incorporation of RE resources at the uncertain condition.

### 1.4 Paper layout

The remainder of the paper is structured as follows: Section 2 ‘Uncertainty Modeling’ describes the uncertainty of RE resources and load demand. Section 3 ‘Problem Formulation’ describes the objective function and the problem constraints. Section 4 ‘Artificial Gorilla Troops Optimizer’ presents an overview of GTO that has been applied on the problem. Section 5 ‘Simulation results and discussion’ introduces the studied cases, the effectiveness of used algorithm and the results’ comparison. Finally, Section 6 ‘Conclusion’ outlines the paper’s results.

## 2. Uncertainty modeling

During the operation of power systems including integrated RE generation units, uncertainty can appear in both generation side and load side (referring to forecasting errors and demand response). The proper modelling and analysis of these uncertainties ensure the solution of UC problem to be more reliable and reasonable. The unstable behavior of the solar and wind power and the load demand are statistically described by the application of probability density functions (PDFs). As the probability density functions (PDF) parameters are assumed to be known for the system variables during each time period *t*, so to simulate the uncertainties of RE resources and load demand, Monte Carlo Simulation (MCS) method is used to produce scenarios, which are then reduced by application of scenario reduction methods.

### 2.1 Wind speed modeling

Random nature of wind speed which is the key for determining the power produced by a wind turbine. In the UC problem (deterministic model), using predicted wind speed to determine output power from wind turbines is insufficient for practical power system operation. The following is a brief overview for wind speed uncertainty described using PDF:

**Step 1:** We assume that the wind speed has a Weibull distribution [55]. For the wind speed *V*_*t*_ (m/s) at the *t* th time interval, the Weibull distribution can be defined as in (1):

$${f_{V}}^{t}(V)=\frac{k_{t}}{c_{t}}.{\left(\frac{V_{t}}{c_{t}}\right)}^{k_{t}-1}.\exp\left(-{\left(\frac{V_{t}}{c_{t}}\right)}^{k_{t}}\right)$$

$$for\;c_{t}>1\;and\;k_{t}>0$$ (1)

Where k_t_ and c_t_ represent the shape and scale parameter of Weibull distribution at the t th time interval, respectively. They are expressed in (2) and (3):

$$k_{t}={\left(\frac{{\sigma_{t}}^{V}}{{\mu_{t}}^{V}}\right)}^{-1.086}$$ (2)

$$c_{t}=\frac{{\mu_{t}}^{V}}{\Gamma (1+\frac{1}{k_{t}})}$$ (3)

Where *μ*_*t*_^*V*^ and *σ*_*t*_^*V*^ are mean and standard deviation of wind speed at time segment *t*.*Γ* gives the Gamma function.

After that a large set of scenarios are created using MCS technique which relies upon random variables to create a huge set of predicted scenarios [15, 56]. The produced *N* scenarios can be represented in 24 hours by a matrix *X*_*N*_ as in (4):

$$X_{N}=[\begin{array}{cccc} S_{1,1} & S_{2,1} & \cdots & S_{T,1}\\ S_{1,2} & S_{2,2} & \cdots & S_{T,2}\\ \vdots & \vdots & \cdots & \vdots \\ S_{1,N} & S_{2,N} & \cdots & S_{T,N} \end{array}]$$ (4)

**Step 2**: Using a backward reduction algorithm (BRA) [57, 58], the number of generated scenarios in step 1 is minimized effectively to a smaller scale. They are scenario-based strategies intended towards obtaining a smaller number of scenarios that are relatively close to the original system. So, a reduction in the scenarios numbers is achieved with various probabilities are achieved, and an accurate estimation of the uncertain performance of the system is maintained.

### 2.2 Solar irradiance modeling

Solar energy is another form of RE that gain its intermediated nature due to continuous variations of the solar irradiance. For modelling Solar power uncertainty, we assume that the solar irradiance has a Beta distribution [59]. The beta distribution for solar irradiance *SR*_*t*_ (kW/m^2^) at the *t* th time interval can be defined as in (5):

$${f_{SR}}^{t}(SR)=\frac{\Gamma (\alpha_{t}+\beta_{t})}{\Gamma (\alpha_{t}).\Gamma (\beta_{t})}.{\left({SR}_{t}\right)}^{\alpha_{t}-1}.{\left(1-{SR}_{t}\right)}^{\beta_{t}-1}$$

$$for\;\alpha_{t}>0\;and\;\beta_{t}>0$$ (5)

Where *α*_*t*_ and *β*_*t*_ represent the shape parameters during time segment *t*; *Γ* is the Gamma function. The shape parameters of Beta distribution at the t th time interval are expressed as in (6) and (7):

$$\beta_{t}=(1-{\mu_{t}}^{SR}).(\frac{{\mu_{t}}^{SR}(1+{\mu_{t}}^{SR})}{{\left({\sigma_{t}}^{SR}\right)}^{2}}-1)$$ (6)

$$\alpha_{t}=\frac{{\mu_{t}}^{SR}.\beta_{t}}{\left(1-{\mu_{t}}^{SR}\right)}$$ (7)

Where *μ*_*t*_^*SR*^ and *σ*_*t*_^*SR*^ are the mean and SD of solar irradiance at time interval *t*.

The same two step procedure applied to represent wind speed uncertainty is used to represent solar irradiance uncertainty.

### 2.3 Load demand modeling

In order to simulate the uncertainty of load demand in the power system, also PDF is used, which can be defined as in (8):

$${PDF}_{L}({P^{t}}_{L})=\frac{1}{\sqrt{2\pi {\sigma^{t}}_{L}}}exp\left[-\frac{{{{(P}^{t}}_{L}-{\mu^{t}}_{L})}^{2}}{2{{\sigma^{t}}_{L}}^{2}}\right]$$ (8)

where PDF_L_ represents the PDF of load demand; *P*^*t*^_*L*_ represents the apparent power of load demand; *μ*^*t*^_*L*_ represents the mean value of the load at time *t*; *σ*^*t*^_*L*_ represents the SD of the load at time *t*.

We consider that the load has a normal distribution [15]. The mean and standard deviations (*μ*^*t*^_*L*_ and *σ*^*t*^_*L*_) are known for each period of time *t*.

## 3. Problem formulation

### 3.1 Objective function

UC’s aim is to diminish the system’s operating cost by predicting the optimal operation schedule and the produced power of the existing generating units while meeting a number of constraints. The integration of RE resources with the consideration of uncertainty complicates the optimization of the UC objective function as additional constraints must be added. In this paper, the operating costs of RE units is neglected as they consume no fuel so, the system total operating cost to be minimized depend on Fuel cost (costs of power generation), and starting-up costs of typical thermal units. Using scenario analysis for uncertainty modelling, the objective function of the UC is optimized to analyze the system’s overall operating cost under uncertainty of RE resources and load demand. Mathematically, it is expressed as in (9):

$$Min\;TC=\sum_{t=1}^{T}\sum_{i=1}^{N}{U^{t}}_{i}\times {{FC}^{t}}_{i}+{{SC}^{t}}_{i}$$ (9)

Where *TC* is the total operating cost; *U*^*t*^_*i*_ is represents the operating state of the unit *i* at time *t*; *FC*^*t*^_*i*_ represents the fuel cost of unit i at time *t*; *SC*^*t*^_*i*_ represents the start-up cost of unit i at time *t*; *t* gives the time horizon for a set of *T*; *i* is the thermal power units for a group of *i*.

#### 3.1.1 Fuel cost

The cost of fuel can be defined as a quadratic equation in case of not considering the valve point effect (VPE). It is represented as in (10):

$${{FC}^{t}}_{i}=a_{i}+b_{i}\times P_{i}(t)+c_{i}\times {P^{2}}_{i}(t)$$ (10)

Where *a*_*i*_,*b*_*i*_ and *c*_*i*_ give the coefficients of the fuel cost of *i*^*th*^ unit; *P*_*i*_(*t*) represents the generated power from unit *i* at time *t*.

To have more nonlinearity in the evaluation in the operating cost, the VPE is considered in which the fuel cost function is expressed in a higher order equation. A sinusoidal term is added to the quadratic function [60]. It is represented as in (11):

$${{FC}^{t}}_{i}=a_{i}+b_{i}\times P_{i}(t)+c_{i}\times {P^{2}}_{i}(t)+|d_{i}\times \sin\left(e_{i}\times (P_{i_{min}}-P_{i}(t)\right)|$$ (11)

#### 3.1.2 Start-up cost

It represents the expense that a thermal unit incurs when it starts up. Before they can be used, thermal units must first be "warmed up." The cost of the warming up procedure influences the overall operating cost. The thermal unit restarting cost is estimated by the amount of time it has been turned off. The costs of starting the thermal units vary depending on the unit properties. The starting up cost of unit *i* is defined in (12):

$${{SC}^{t}}_{i}=\left\{ \begin{array}{l} {SC}_{i_{hot}}\to {MDT}_{i}\leq T_{{OFF}_{i}}\leq {MDT}_{i}+T_{{cold}_{i}}\\ {SC}_{i_{cold}}\to T_{{OFF}_{i}}>{MDT}_{i}+T_{{cold}_{i}} \end{array}\right.$$ (12)

Where ${SC}_{i_{hot}}$ represents the hot start-up cost of unit *i*; ${SC}_{i_{cold}}$ gives the cold start-up cost of unit *i*; *MDT*_*i*_ gives the minimum off time of unit *i*; $T_{{OFF}_{i}}$ represents time that unit *i* has been consistently off; $T_{{cold}_{i}}$ represents the needed time for unit *i* to become completely cool.

### 3.2 Constraints

#### 3.2.1 Power equilibrium constraint


$$\sum_{i=1}^{N}{U_{i}(t)\times P}_{i}(t)=L_{net}(t)$$ (13)

Where *L*_*net*_(*t*) represents the net load at time *t*.


$$L_{net}(t)={P^{t}}_{L}-P_{W,out}(t)‐P_{PV,out}(t)$$ (14)

Where *P*_*L*_^*t*^ gives the system load demand at time *t*; *P*_*W*,*out*_(*t*) represents the wind turbine produced power at time *t*; *P*_*PV*,*out*_(*t*) gives the solar unit produced power at time *t*.

The net load refers to the gap between the served load and the total capacity generated by the renewable energy resources (wind turbines and solar units). In other words, it represents the power that will be provided by the thermal units when RESs are in service.

#### 3.2.2 Up/down spinning reserve

The up-spinning reserve participates in load forecasting errors, unplanned generator outages, a drop in wind speed and/ or solar irradiance. The sudden rise in wind speed and/ or solar irradiance is supported by the down spinning reserve. This constraint is given in (15):

$$\sum_{i=1}^{N}U_{i}(t)\times P_{i_{max}}+P_{W,out}(t)+P_{PV,out}(t)\geq {SR}^{t}+{P^{t}}_{L}$$

$$\sum_{i=1}^{N}U_{i}(t)\times P_{i_{min}}+P_{W,out}(t)+P_{PV,out}(t)<{SR}^{t}+{P^{t}}_{L}$$ (15)

Where $P_{i_{max}}$ and $P_{i_{min}}$ give the upper and lower generation limit of unit *i*, respectively; *SR*^*t*^ represents the system’s spinning reserve needs at time *t*.

#### 3.2.3 Output power limits for thermal generating units

$$P_{i_{min}}\leq P_{i}(t)\leq P_{i_{max}}$$ (16)

#### 3.2.4 Minimum up/down time constraints

- Minimum up time constraint

This constraint is reflected in the fact that a unit cannot be shut down immediately after it has been switched on and it can be mathematically defined as in (17):

$$T_{{ON}_{i}}\geq {MUT}_{i}$$ (17)

Where $T_{{ON}_{i}}$ represents time that unit *i* has been consistently on; *MUT*_*i*_ is the minimum up time of unit *i*.

- Minimum down time constraint

This constraint is reflected in the fact that a unit cannot be restarted immediately after it has been switched off and it can be mathematically defined as in (18):

$$T_{{OFF}_{i}}\geq {MDT}_{i}$$ (18)

#### 3.2.5 Ramping capacity constraint for thermal generating units

The output power generated by thermal units should be increased or decreased only by a certain amount described as ramping capacity of the unit. It can be expressed mathematically as in (19):

$${-P}_{{ramp}_{i}}\leq P_{i}(t)-P_{i}(t-1)\leq P_{{ramp}_{i}}$$ (19)

Where *P*_*i*_(*t*−1) represents the generated power from unit *i* at time (*t*−1); $P_{{ramp}_{i}}$ gives the ramp-up power capacity of unit *i*.

#### 3.2.6 Production limits of wind turbine

A wind turbine produced power is expressed based on the wind speed as follows:

$$P_{W,out}=\left\{ \begin{array}{ccc} 0 & for & V_{w}<V_{in}\;and\;V_{w}>V_{out}\\ P_{R}(\frac{V_{w}-V_{in}}{V_{R}-V_{in}}) & for & \left(V_{in}\leq V_{w}\leq V_{R}\right)\\ P_{R} & for & \left(V_{R}<V_{w}\leq V_{out}\right) \end{array}\right.$$ (20)

Where P_R_ represents the base produced power from the turbine; V_R_ gives the base wind speed; V_in_ represents the cut-in speed; V_out_ represents the cut-out speed; V_w_ represents the actual speed of the wind.

#### 3.2.7 Production limits of solar unit

A solar unit produced power is expressed based on the solar irradiance according to Eq (21):

$$P_{PV,out}=\;\;\left\{ \begin{array}{c} P_{sr}(\frac{{G_{s}}^{2}}{G_{std}\times X_{c}})\;for\;0<G_{s}\leq X_{c}\\ P_{sr}(\frac{G_{s}}{G_{std}})\;for\;G_{s}\geq X_{c} \end{array}\right.$$ (21)

Where *P*_*sr*_ is the rated produced power from the solar unit; *G*_*s*_ represents the solar irradiance; *G*_*std*_ represents the solar irradiance for standard environment conditions.

## 4. Artificial gorilla troops optimizer

### 4.1 Inspiration

Artificial Gorilla Troops Optimizer (GTO) is an innovative optimization technique which simulates natural lifestyle, social interaction, and immigration of the gorilla [52]. The gorilla group is led by a dominant silverback gorilla, and all males and females in the group obey the silverback gorilla. Black backs, or young male gorillas, obey silverbacks and serve as the group’s backup guards. Sometimes, female gorillas travel to new groups. GTO method uses Five distinct operators for exploration and exploitation phases depend on the habits of gorilla. Three operators are used to control the exploration phase. The first operator is the relocation to unknown areas in order to expand the exploration of GTO. The second one is the travelling towards other gorillas, whereas the third operator is the movement of the groups to known locations which considerably improves the GTO’s capabilities to seek for alternative optimization areas. In the exploitation phase, two approaches are employed. The first one depends on silver back tracking, whereas the second one depends on adult female mobility. The optimization area of the GTO has three solutions (*X*,*GX*, and the silverback). *X* denotes the gorillas’ position. *GX* denotes the gorilla candidate position, whereas the silverback is the best solution. There is a single silverback in the overall population whenever it comes to the quantity of search agents used for optimization operations.

The three sorts of solutions: *X*,*GX*, and silverback, closely resemble gorilla social behaviour in the wild. Gorillas try to find new food areas or build a powerful and equitable group. In each iteration in the GTO technique, solutions are developed and known as *GX* and replaced if another solution with better value is found. Otherwise, it will remain as *GX*. The GTO algorithm employs several strategies for optimization operations, that are discussed below.

### 4.2 Exploration phase

All gorillas are regarded as possible solutions in the GTO algorithm, and the optimal solution at each optimization operation step is referred to as a silverback gorilla. As previously stated, the exploration phase is based on three mechanisms: gorilla movement to new unknown positions, gorilla travel to known positions, and gorilla migration to other gorillas. An operator called *P*, that has a range of 0 to 1, can be used to adjust the crossover between the three mechanisms. When *rand*<*P*, the first mechanism is selected. If the *rand*<0.5, the gorilla adjusts its position to a known one. If *rand*>0.5, the technique of travelling towards other gorillas is chosen. Mathematically, the three mechanisms utilized in the exploration phase can be represented as follows:

$$GX(t+1)=\left\{ \begin{array}{l} (ub-lb)\times r_{1}+lb,\;rand<p\\ (r_{2}-C)\times X_{r}(t)+L\times H,\;rand\geq 0.5\\ X(i)-L\times (L\times (X(t)-GX_{r}(t))+r_{3}\times (X(t)-GX_{r}(t))),rand<0.5\; \end{array}\right.$$ (22)

$$GX(t+1)=(ub-lb)\times r_{1}+lb\;If\;\mathrm{rand}<p$$ (23)

$$GX(t+1)=(r_{2}-C)\times X_{r}(t)+L\times H\;If\;\mathrm{rand}\geq 0.5$$ (24)

$$GX(t+1)=X(i)-L\times (L\times (X(t)-GX_{r}(t))+r_{3}\times (X(t)-GX_{r}(t)))\;If\;\mathrm{rand}<0.5$$ (25)

Where *GX*(*t*+1) represents the vector of the candidate solution in the next iteration. *X*(*t*) gives the vector of current gorilla position. *ub* and *lb* give the upper and lower limits of the control elements. *r*1,*r*2,*r*3 and *rand* are randomized numbers in the range of [0–1]. *X*_*r*_ represents one of the group’s gorillas, picked at random from the entire population, as well as *GX*_*r*_ is the position vector of one of the gorilla candidates picked randomly. *C*,*L* and *H* are operators that are represented mathematically as shown below:

$$C=F\times \left(1-\frac{iter}{maxiter}\right)$$ (26)

$$F=cos(2\times r_{4})+1$$ (27)

L=C×l (28)

Z=[−C,C] (29)

H=Z×X(t) (30)

Where *iter* represents the current iteration and maxiter denotes the maximum number of iterations. *r*_4_ represents a randomized number between 0 and 1. *l* is a random number varies from -1 to 1. *Z* is a randomized number that locates between −*C* and *C*.

Finally, a group construction process is carried out, during which the cost of all solutions is evaluated, and the best solution is remembered. As a result, the best solution created during this phase is referred to as a silverback.

### 4.3 Exploitation phase

The silverback gorilla is the head of the group, makes all the choices, guides the gorillas to sources of food, controls the group’s movements and accounts for the group’s security. The silverback gives orders to the bunch of gorillas, and they all comply. On the other hand, the silverback gorilla might become feeble and old, finally die, and the group’s black-back gorilla may take over as a leader, or the silverback gorilla may be fought off and managed by other male gorillas. As stated with the two strategies previously mentioned in the exploitation phase, the other gorillas can select to either obey the silverback one or struggle for adult females. The *W* value, which is a preset value and the value of C shown in (26) are used to changeover between the two motions.

#### 4.3.1 Follow the silverback

This technique is the choice if *C*≥*W* and the positions of gorillas are updated by obeying the silverback gorilla as in (31):

$$GX(t+1)=L\times M\times (X(t)-X_{\mathrm{silverback}})+X(t)$$ (31)

Where *X*_*silverback*_ represents the position of the silverback gorilla. *X*(*t*) represents the gorilla location. *L* is determined by (28) and *M* can be determined as in (32).


$$M={\left({\left|\frac{1}{N}\sum_{i=1}^{N}\;GX_{i}(t)\right|}^{g}\right)}^{\frac{1}{8}}$$ (32)

$$g=2^{L}$$ (33)

Where *GX*_*i*_(*t*) gives candidate position for each gorilla during iteration *t*.*N* is the overall number of gorillas.

#### 4.3.2 Competition for adult females

This technique is the choice if *C*<*W* and the adjustment of gorillas’ positions are determined based on a struggle for adult females. This mechanism is represented mathematically according to (34):

$$GX(i)=X_{\mathrm{silverback}}-(X_{\mathrm{silverback}}\times Q-X(t)\times Q)\times A$$ (34)

$$Q=2\times r_{5}-1$$ (35)

A=β×E (36)

$$E=\left\{ \begin{array}{c} N_{1},\;rand\geq 0.5\\ N_{2},\;rand<0.5 \end{array}\right.$$ (37)

Where *Q* simulates the impact force. *β* represents a preset parameter. *r*_5_ represents a random number in range of 0 and 1. *E* represents the violence impact on the solutions’ dimensions and can be calculated as given in (37). In (37) if *rand*≥0.5, The value of *E* is equivalent to random number in the normal distribution and the problem’s dimensions, but if rand < 0.5, *E* is equivalent to a random number in the normal distribution.

Finally, a group construction process is carried out during which the cost of all solutions is evaluated, and the best solution achieved in the entire population is recognized as a silverback.

**Fig 1:**
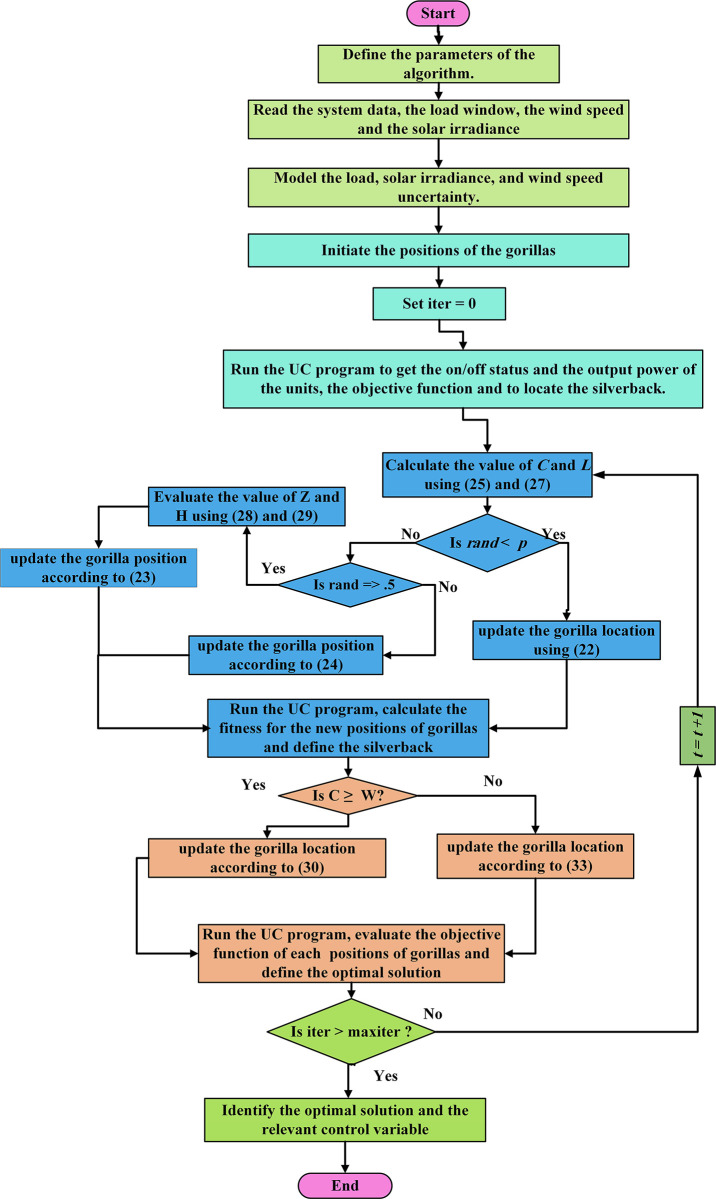
Flow chart of applying the GTO in solving stochastic UC problem.

Application of the GTO for handling the SUC problem with integration of RE sources is presented in Fig 1.

## 5. Simulation results

In this section, the UC problem has been studied from various perspectives through investigation of three cases that represent various arrangements of power sources. First case studies the DUC with system of ten thermal units. This case is used to test the ability of the GTO to handle the UC problem. It serves as a validation for the GTO to be applied for the UC problem. In the second case, the SUC is investigated at uncertain load demand without integration of RE resources. In the third case, RE units (wind and solar) are introduced to the system of the second case to investigate the effect of incorporation of the RE units and their uncertainties along with the uncertainty of the load side on the solution of the UC problem and on the system’s performance and economics. The simulations have been run in MATLAB 2020a on a PC with an Intel Core i7 processor, 8 GB RAM, and a Microsoft Windows operating system.

### 5.1 Case 1: Solution of UC with deterministic load and without RE units

In this case, the system has only 10 thermal units while considering deterministic load demand. The scheduling period is a one day. According to the power system dependability measurement, the spinning reserve required in order to face sudden changes in generation and/or load should be 10% of the load. Table 1 gives the forecasted load data over 24-hour horizon [28]. The thermal generating units data are presented in Table 2 [61]. The solution of this case is used to test the efficacy of GTO in handling the UC problem, so the findings are compared to number of the well-known algorithms. This case is studied with and without considering the valve point effect (VPE) to test the efficacy of GTO in handling the UC with the nonlinearity properties of fuel cost function.

**Table 1:** Forecasted load data.

**Time**	**1**	**2**	**3**	**4**	**5**	**6**	**7**	**8**	**9**	**10**	**11**	**12**
**Forecasted load**	700	750	850	950	1000	1100	1150	1200	1300	1400	1450	1500
**Time**	**13**	**14**	**15**	**16**	**17**	**18**	**19**	**20**	**21**	**22**	**23**	**24**
**Forecasted load**	1400	1300	1200	1050	1000	1100	1200	1400	1300	1100	900	800

**Table 2:** The data for the10-thermal units.

Unit	a ($/h)	b ($/MWh)	c ($/MW^2^h)	$P_{i_{max}}$ (MW)	$P_{i_{min}}$ (MW)	$T_{{cold}_{i}}$ (hr)	${SC}_{i_{hot}}$ ($)	${SC}_{i_{cold}}$ ($)	MUT_i_ (hr)	MDT_i_ (hr)	initial state (hr)
**Unit 1**	1000	16.19	0.00048	455	150	5	4500	9000	8	8	8
**Unit 2**	970	17.26	0.00031	455	150	5	5000	10000	8	8	8
**Unit 3**	700	16.6	0.002	130	20	4	550	1100	5	5	-5
**Unit 4**	680	16.5	0.00211	130	20	4	560	1120	5	5	-5
**Unit 5**	450	19.7	0.00398	162	25	4	900	1800	6	6	-6
**Unit 6**	370	22.26	0.00712	80	20	2	170	340	3	3	-3
**Unit 7**	480	27.74	0.00079	85	25	2	260	520	3	3	-3
**Unit 8**	660	25.92	0.00413	55	10	0	30	60	1	1	-1
**Unit 9**	665	27.27	0.00222	55	10	0	30	60	1	1	-1
**Unit 10**	670	27.79	0.00173	55	10	0	30	60	1	1	-1

#### 5.1.1 solution of UC with deterministic load and without RE units (without the consideration of the valve point effect)

**Fig 2:**
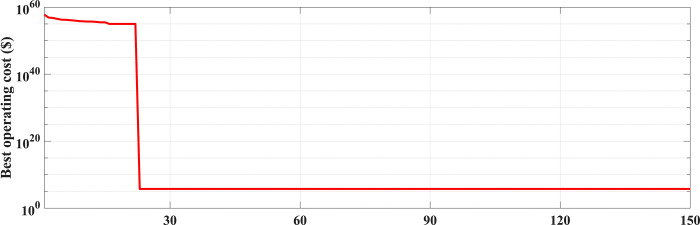
The convergence curve for the GTO handling the DUC (case 1).

**Fig 3:**
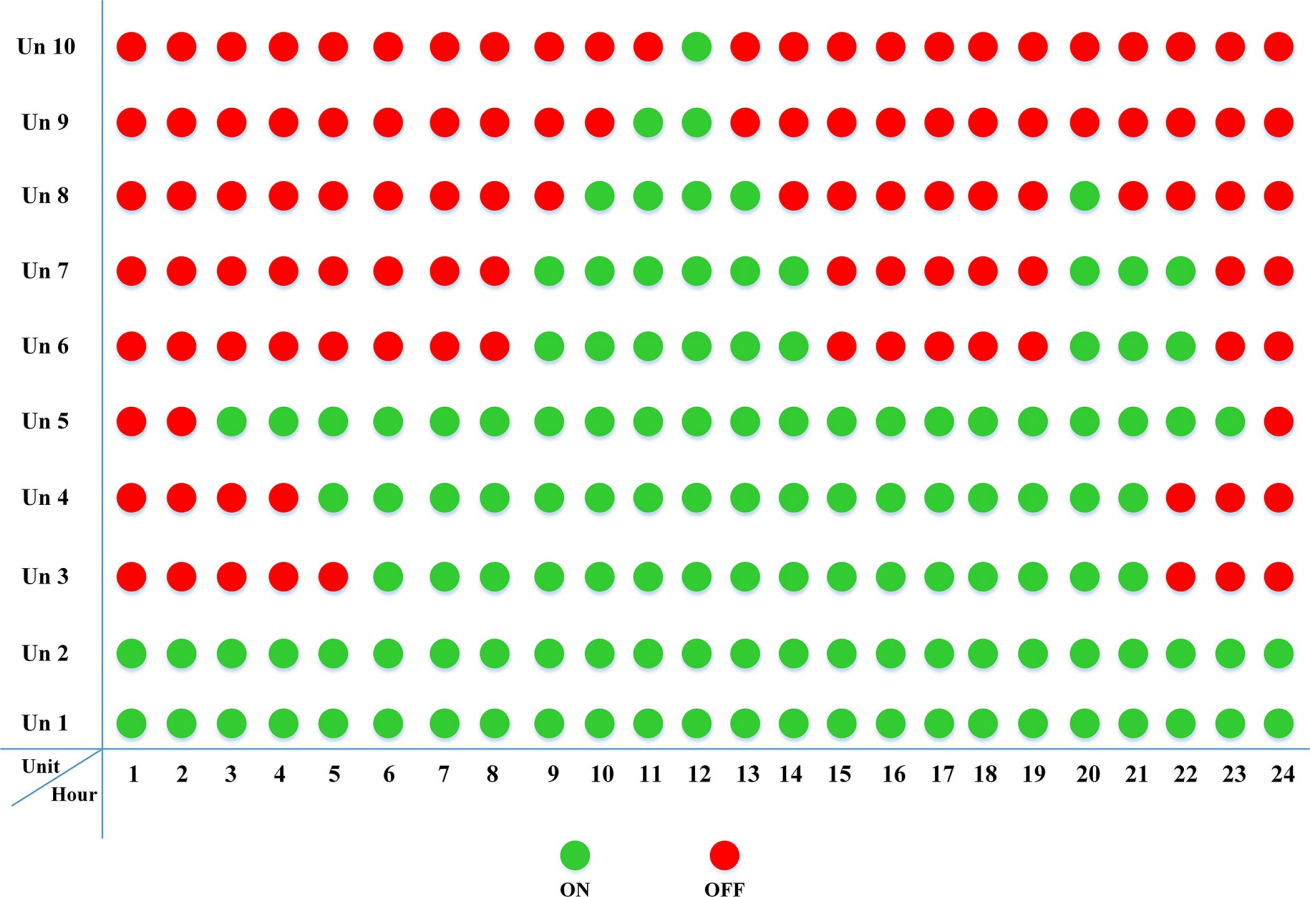
The commitment scheduleing of thermal units of the DUC (case 1).

**Fig 4:**
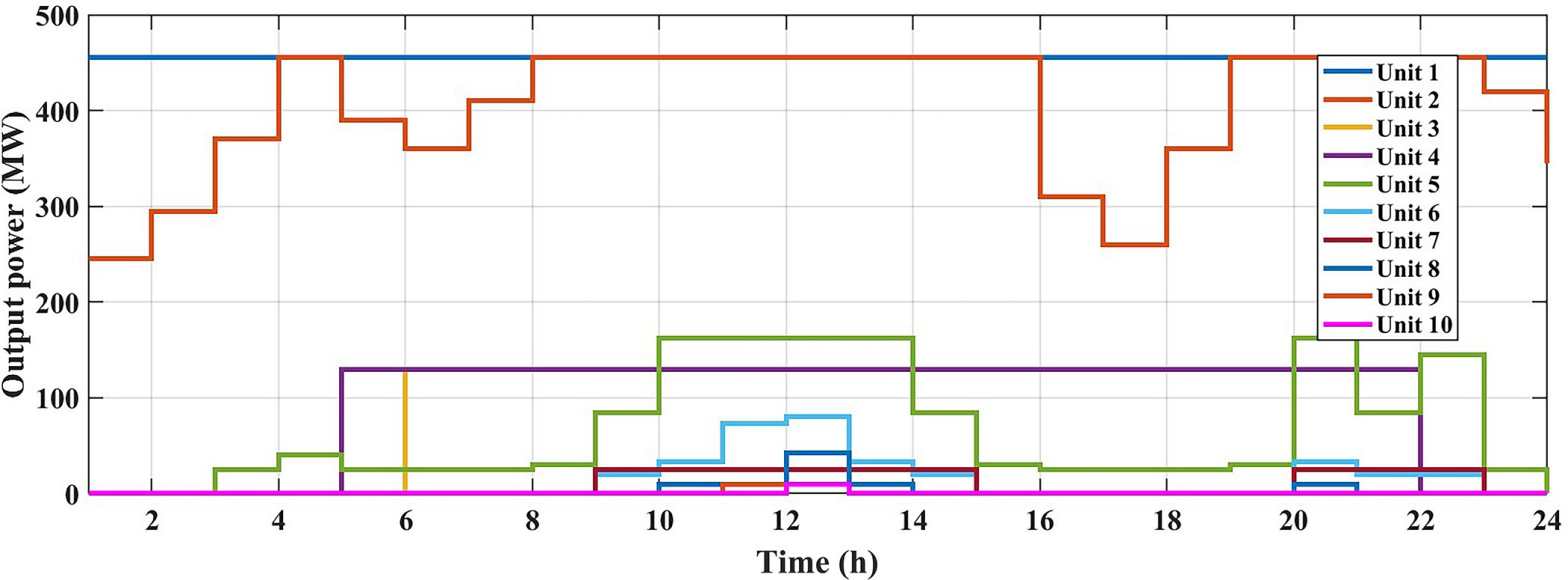
The output powers of the thermal units at the DUC (case 1).

**Fig 5:**
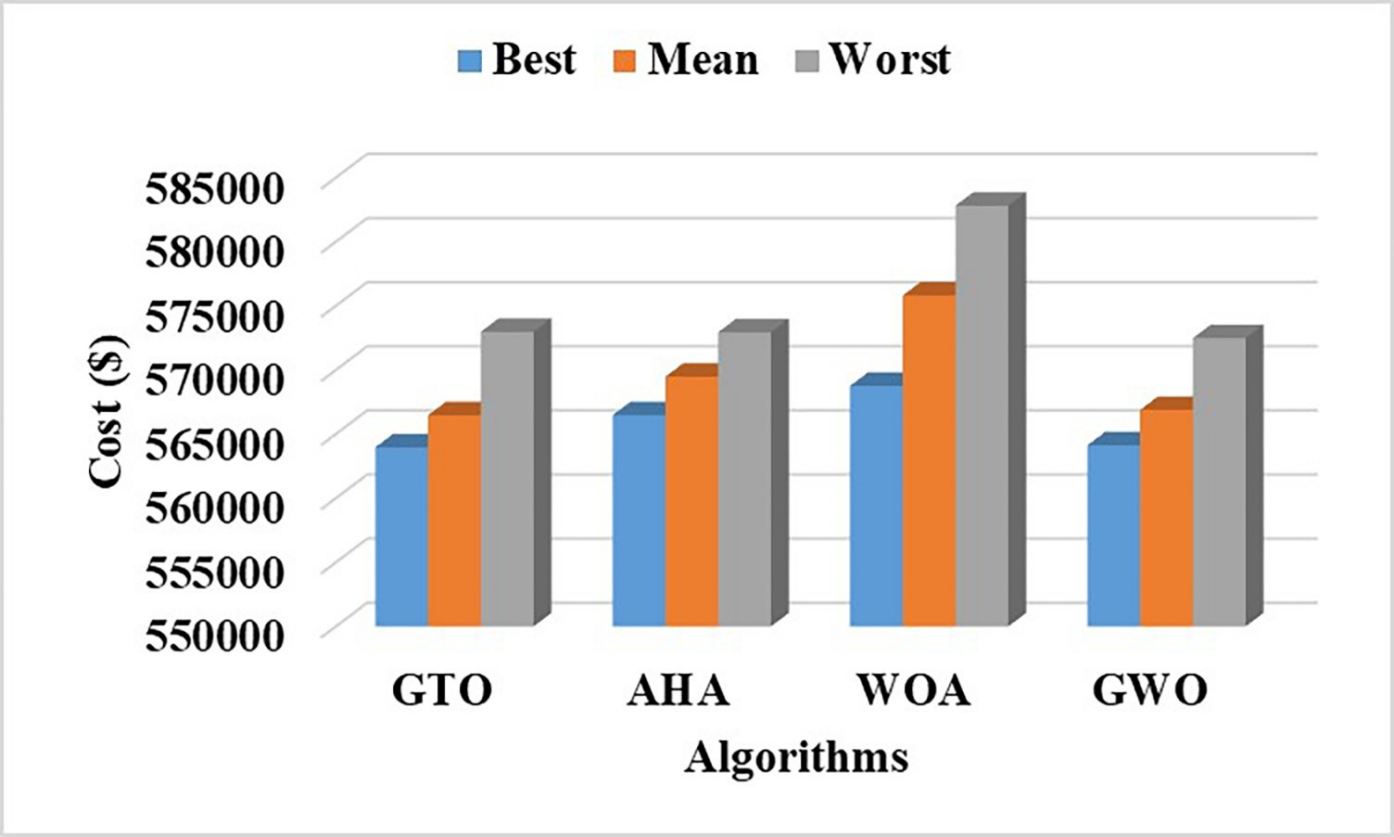
A statistical comparison for cost reduction by different optimizers at DUC.

The fuel cost equation is represented by a quadratic formula. The convergence curve (total operating cost vs iteration process) of GTO is given in Fig 2. It obvious that the GTO has a fast convergence rate. Fig 3 gives the commitment schedule of the thermal units indicated by two colors, green color referred to on status and red one referred to off status. It is shown that unit1 and unit2 are on during the whole scheduling period. Although they have the largest generation coefficients values, but it is ineffective to restart them as their start-up costs are the highest among all units. Fig 4 gives a scheme for the output power obtained from the thermal units. Table 3 compares the overall operating cost acquired by the GTO to the cost reported by various well-known algorithms. The minimum obtained result by the GTO is 563977 ($/day). The minimum obtained results by PSO-GWO [28], BFMO [39], and ABFMO [39] are 565210 ($/day), 585967 ($/day), and 585828 ($/day) respectively. In other words, the cost reduction per day is 0.2181%, 3.7528%, and 3.7299% compared to the GTO. The GTO outperforms the other techniques in tackling the deterministic UC. For more statistical analysis, to prove the effectiveness of the GTO in solving the DUC, the cost obtained by GTO are compared with cost obtained by number of famous algorithms: Grey Wolf Optimizer (GWO), Artificial Hummingbird Algorithm (AHA), and Whale Optimization Algorithm (WOA). Also the obtained results are compared to some experimental studies from the literature. The numerical results are introduced in Table 3. The statistical results are depicted in Fig 5. It shows that the results obtained by GTO gives less operating costs compared to the three algorithms in comparison. This proves the superiority of the GTO in handling the DUC.

**Table 3:** The cost comparison between the GTO and other well-known algorithms without considering VPE (case 1).

Approach	Best operating cost ($)
HASP [26]	564029
SFLA [27]	564769
PSO-GWO [28]	565210
BFMO [39]	585967
ABFMO [39]	585828
AHA	566481
WOA	568762.3
GWO	564131
**GTO**	**563977**

#### 5.1.2 solution of UC with deterministic load and without RE units (with the consideration of the valve point effect)

**Fig 6:**
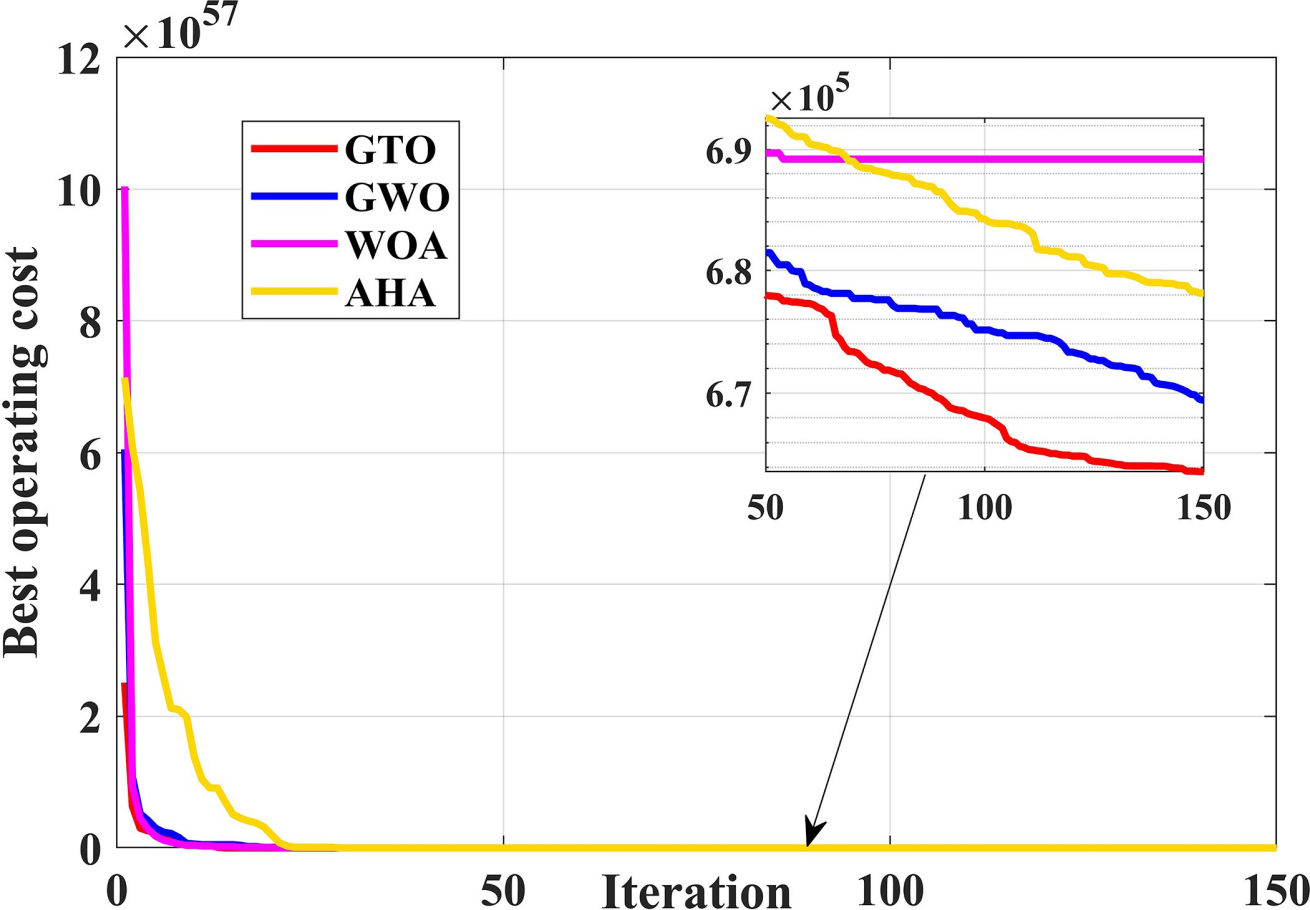
The convergence curves comparison of GTO, GWO, WOA, and AHA with VPE (case 1).

With the consideration of VPE a sinusoidal term is added to the equation of the fuel cost. The data for VPE of ten unit system is taken from [62]. VPE has an impact on the input-output characteristics of generation units, causing the fuel cost to be nonlinear and non-smooth. In most cases, VPE has been considered in the study of economic load dispatch problems. So, in this case of study VPE effect is investigated in the solution of deterministic unit commitment problem. This nonlinearity consideration proves the robustness of GTO against other algorithms. Table 4 gives the obtained results by GTO, GWO, WOA, and AHA. It is obvious that the inclusion of VPE increases the cost, significantly, compared to the cost obtained by GTO without VPE consideration. Also, Table 4 shows that GTO outperforms GWO, WOA, and AHA with less operating cost. A comparison for the convergence curve OF GTO against GWO, WOA, and AHA is depicted in Fig 6. The results demonstrate the superiority of GTO in dealing with the nonlinearity of fuel cost function in the UC problem with best operating cost during the whole iteration process.

**Table 4:** The cost comparison between the GTO and other well-known algorithms with VPE (case 1).

Approach	Best operating cost($)
AHA	678141.05
WOA	689212.89
GWO	669416.19
**GTO**	**663659.48**

### 5.2 Case 2: Solution of UC considering load demand uncertainty without RE units

**Fig 7:**
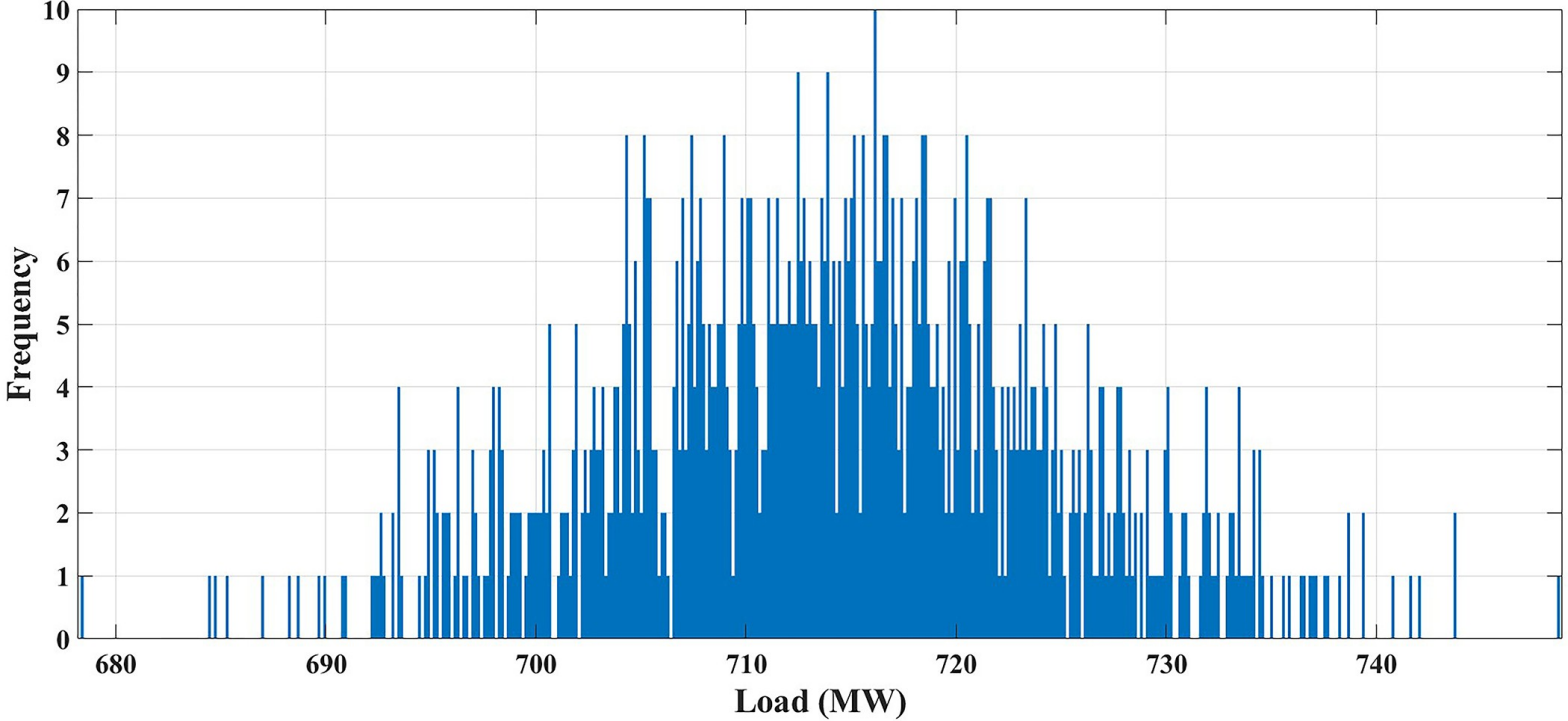
The produced scenarios of the load demand during 12.00–13.00 h by applying 1000 MCSs.

**Fig 8:**
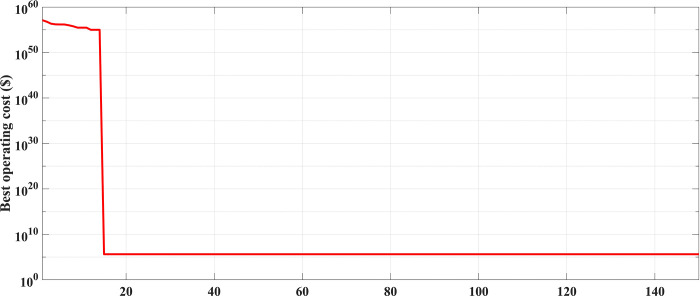
The convergence curve for the GTO handling the SUC at uncertain load demand (case2).

**Fig 9:**
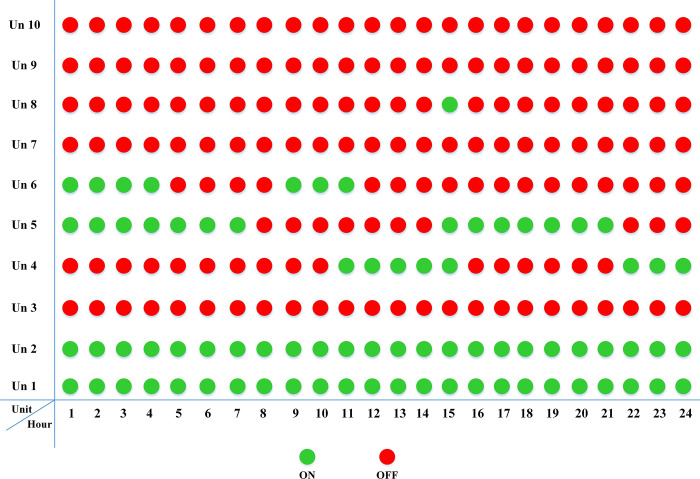
The commitment scheduleing of thermal units of the SUC at uncertainty of the load demand (case2).

**Fig 10:**
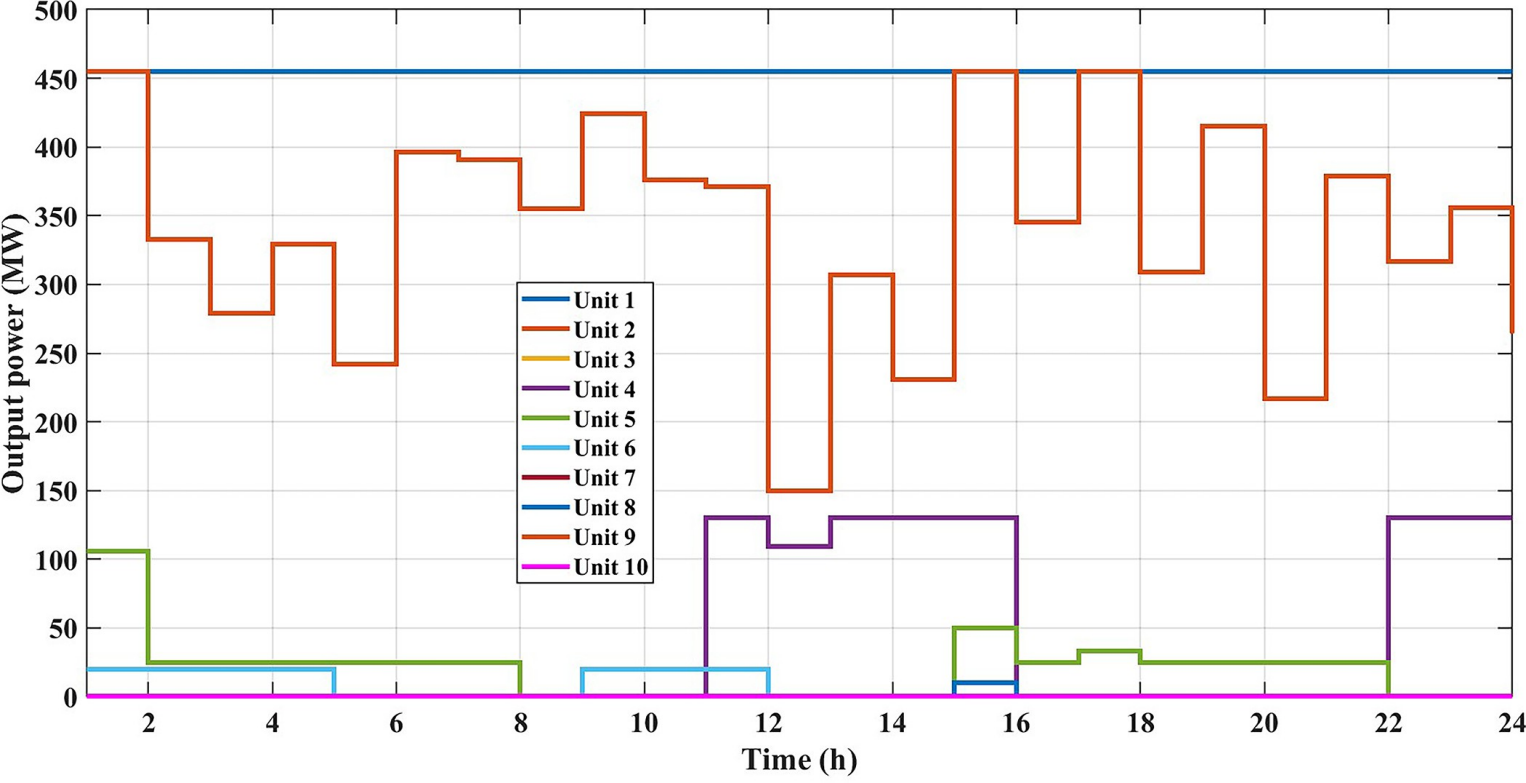
The output powers of the thermal units of the SUC at uncertainty of the load demand (case 2).

In this case, the system has 10 thermal units, but the variability of load demand is considered. The data for 10 units is the same as in case 1. The spinning reserve values are considered to be 10% of the load. Normal distribution was used to represent the load uncertainty. Mean value and standard deviation (SD) are given for each hour [63] and are utilized to create a univariate function to estimate the load value. Using MCS method, in each hour 1000 scenarios are generated to simulate load variations. These scenarios are diminished to ten scenarios which are a good representation of the original system. Fig 7 shows the load demand produced scenarios during 12.00–13.00 h by using the MCS technique. Fig 7 illustrates that during the proposed hour the most repeated load values lay between 710 MW and 720 MW. The convergence curve for the GTO of this case is given in Fig 8. The commitment schedule of thermal units is shown in Fig 9 and is indicated by two colors typically as in case 1. The generated powers from the thermal units are indicated in Fig 10. The operating cost for this case is proposed in Table 5. The minimum obtained cost by the GTO is 416631.12 ($/day). The results show more reliable and realistic solution of UC under load demand uncertainty.

**Table 5:** Costs’ results for case 2.

Test case	Start-up cost ($)	Fuel cost ($)	Operating cost ($)
Case 2	3880	412751.12	416631.12

### 5.3 Case 3: Solution of UC with RE units and with considering uncertainty of the load

**Fig 11:**
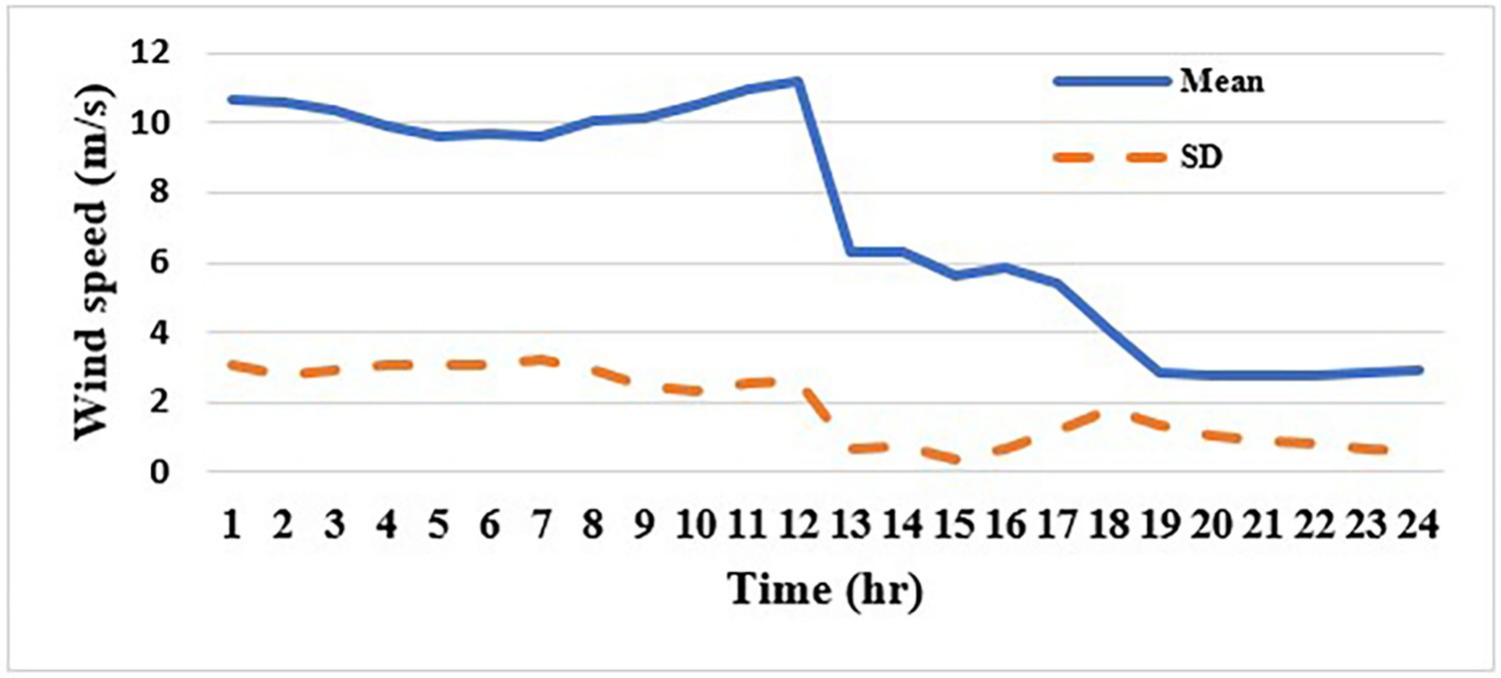
Mean value and SD of wind speed over 24-hr.

**Fig 12:**
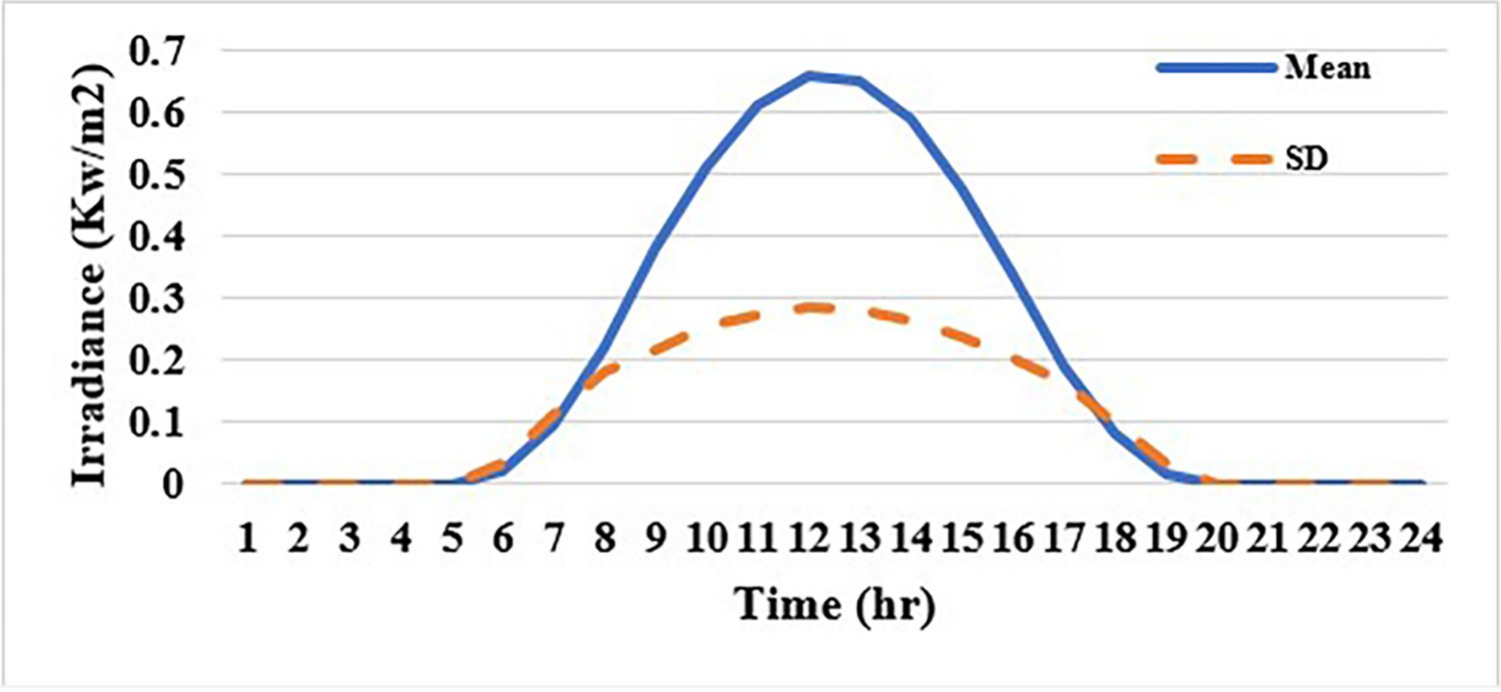
Mean value and SD of solar irradiance over 24-hr.

**Fig 13:**
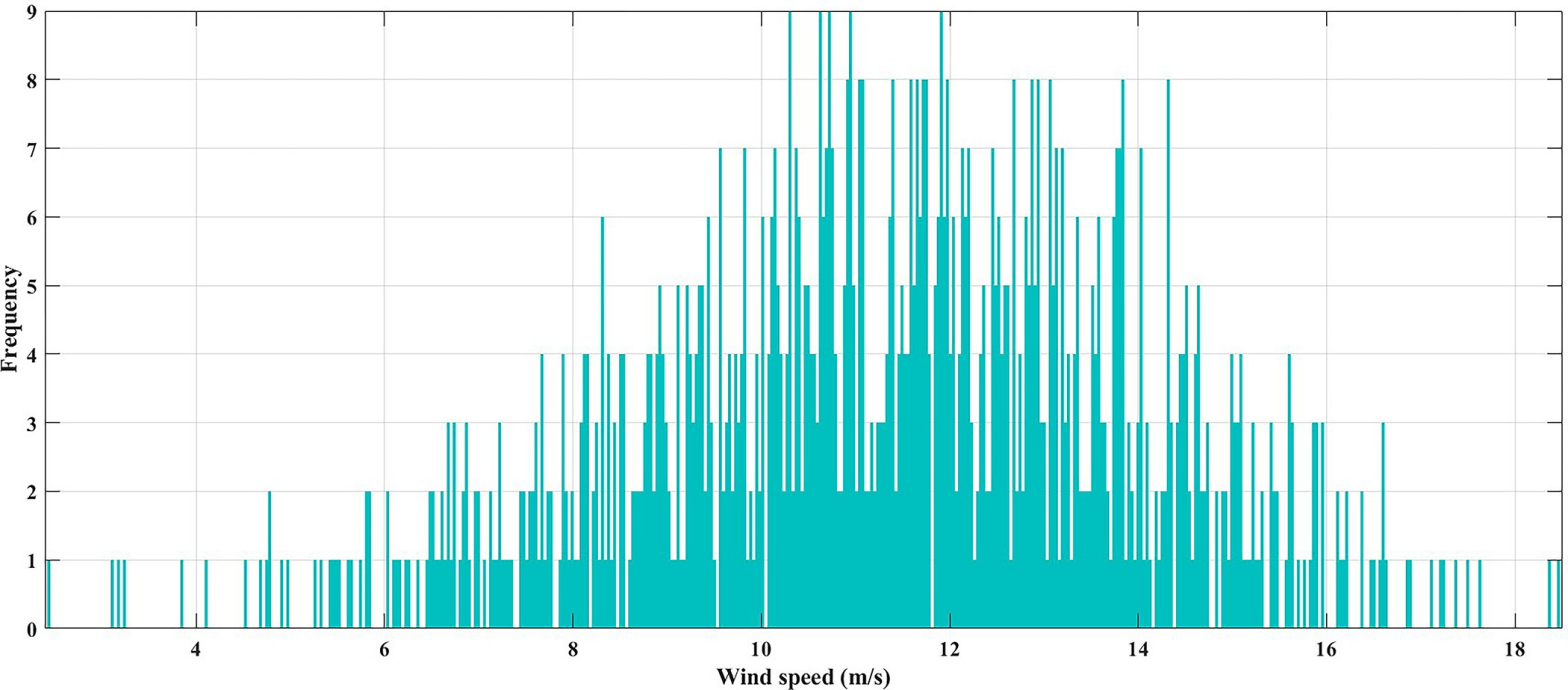
The produced scenarios of the wind speed during 12.00–13.00 h by applying 1000 MCSs.

**Fig 14:**
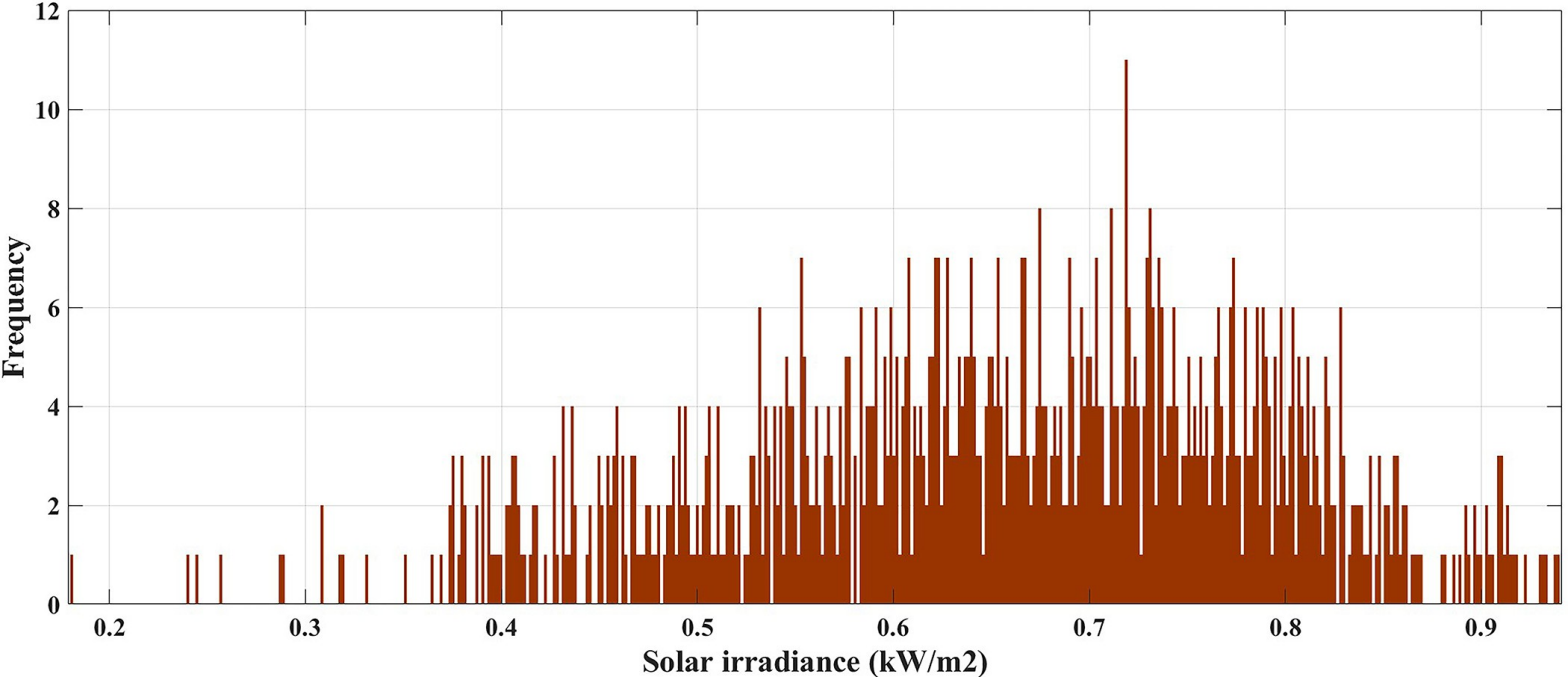
The produced scenarios of the solar irradiance during 12.00–13.00 h by applying 1000 MCSs.

**Fig 15:**
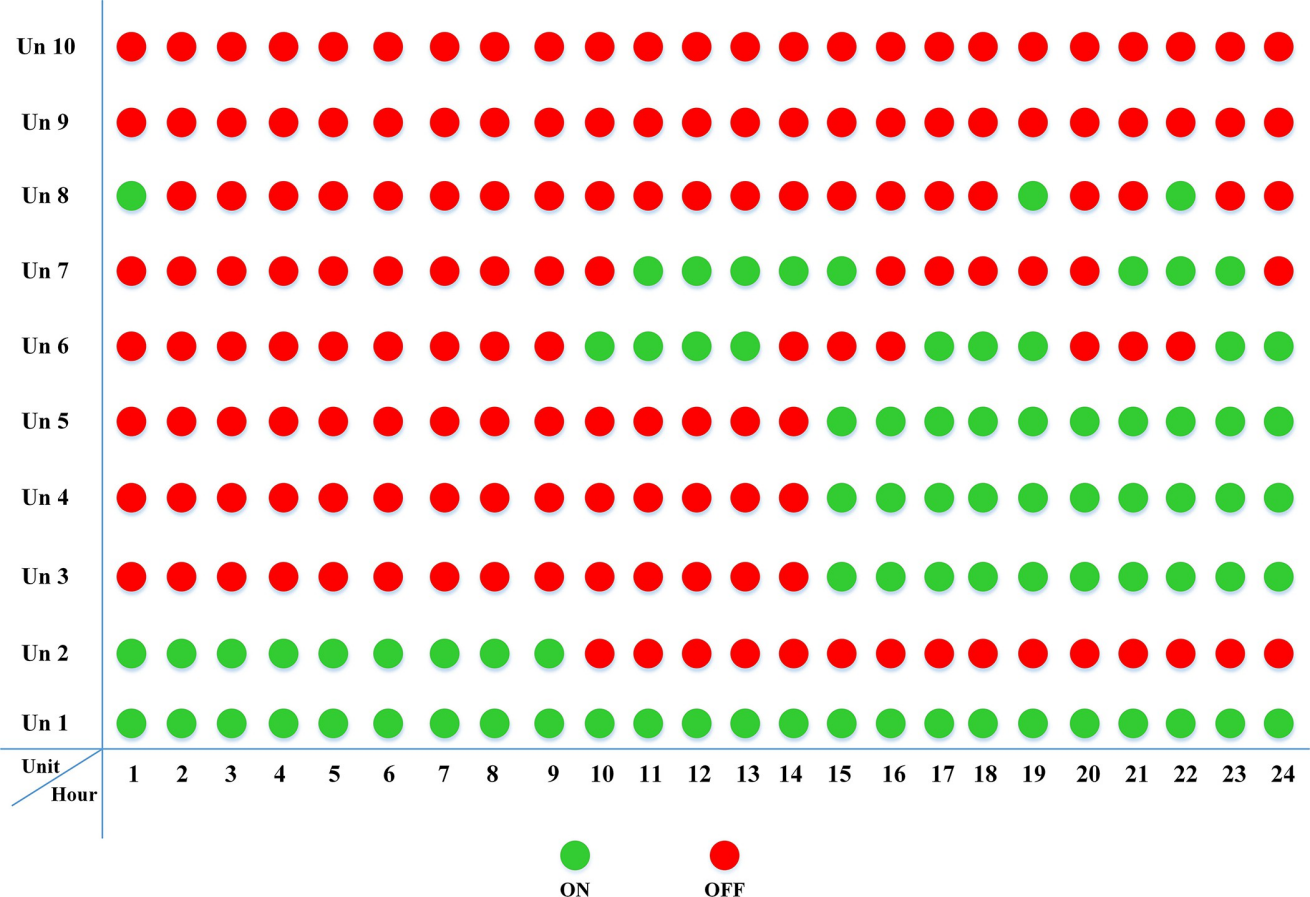
The commitment scheduleing of thermal units of the SUC at uncertainty of the load demand and RE resources (case3).

**Fig 16:**
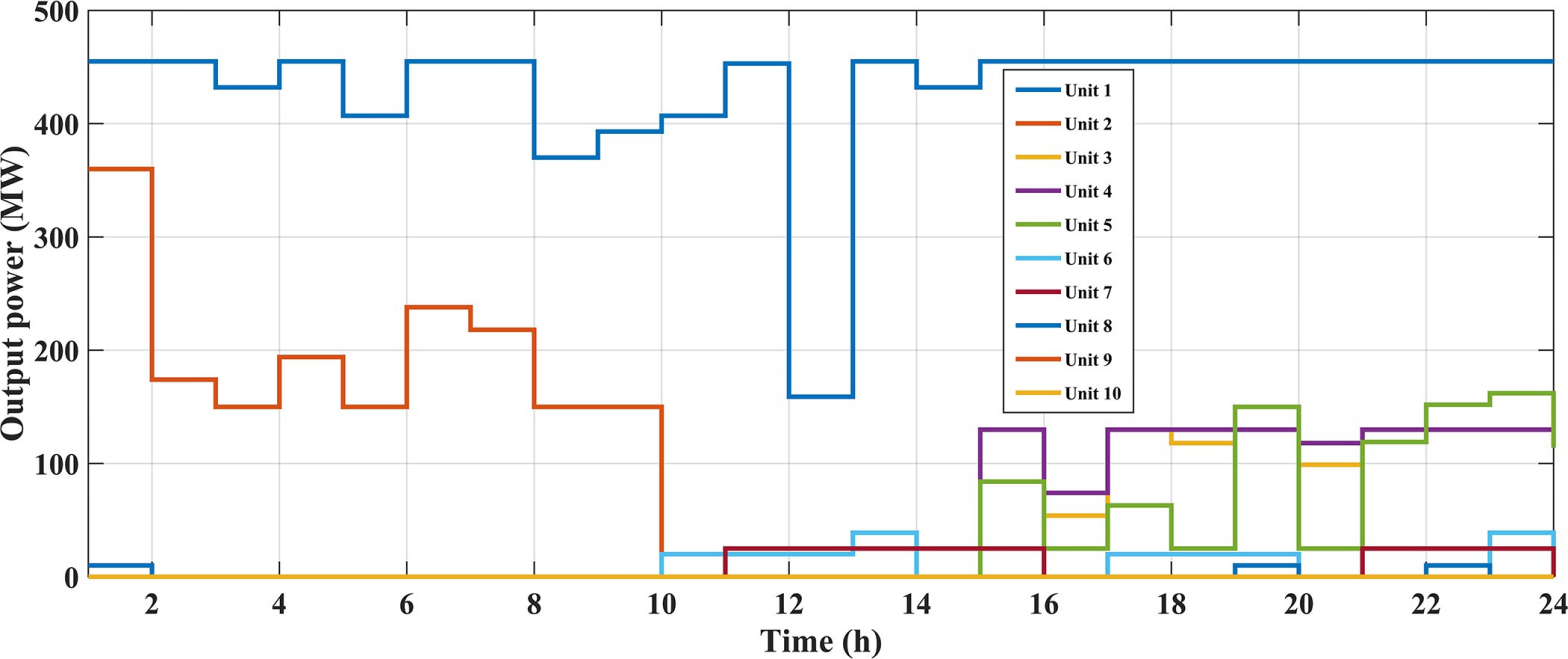
The output powers obtained of the thermal units at the SUC with uncertainty of the load demand and RE resources (case 3).

**Fig 17:**
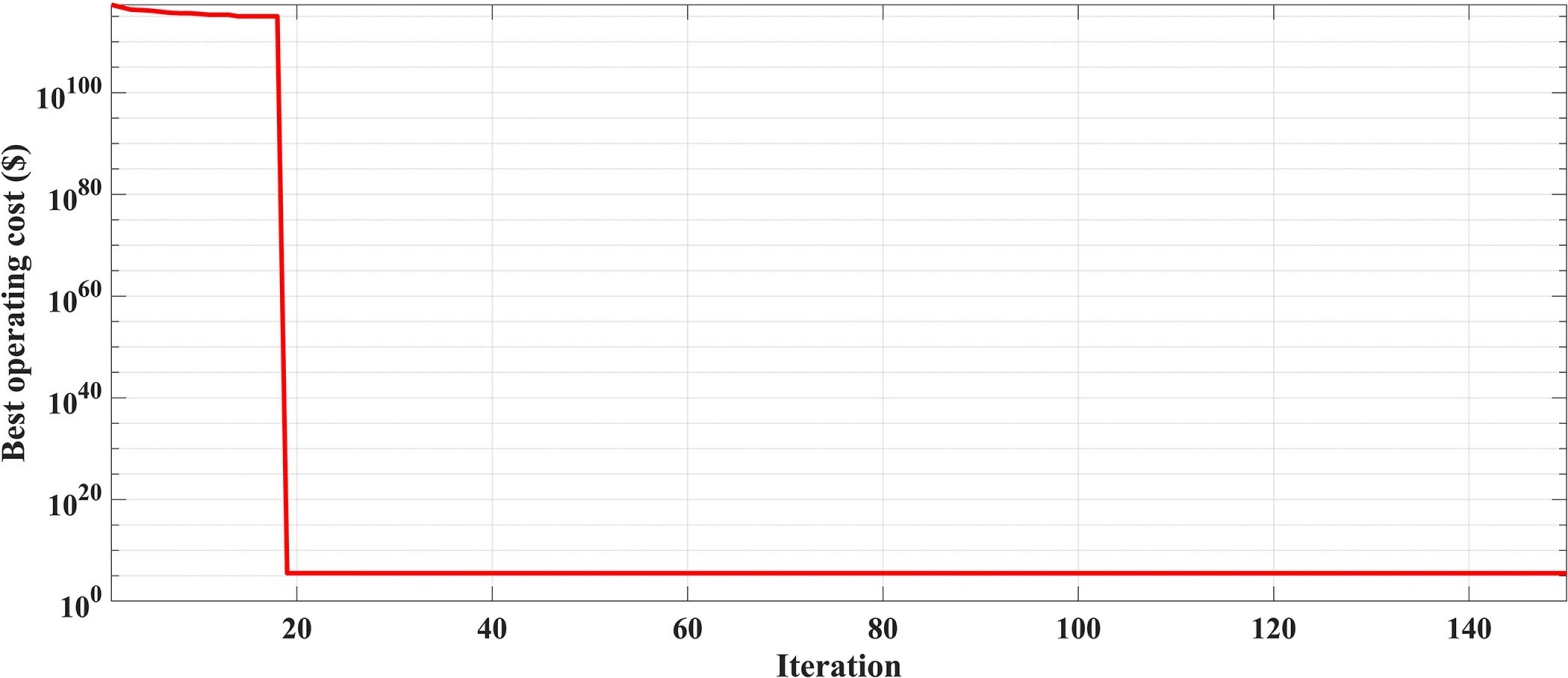
The convergence curve for the GTO handling the SUC at uncertainty of the load demand and RE resources (case3).

**Fig 18:**
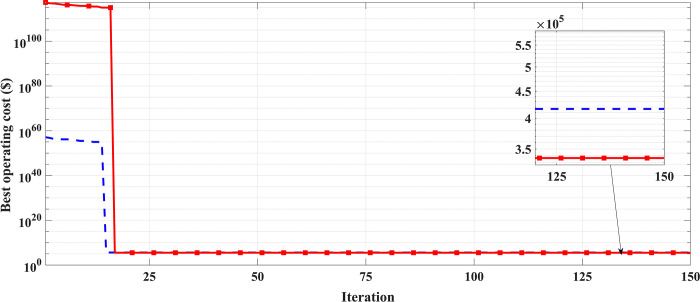
The convergence curves comparison between case 2 and case 3.

In this case, the system was simulated as combination of ten thermal units with a wind farm (six wind turbines) and a solar unit. This system is employed to investigate the impact of integrated RE units and their uncertainties on the UC problem, and as a result the system’s economics. To determine the scale and shape parameters of Weibull distribution of the wind speed variations to calculate the produced power from each turbine and so from the wind farm, the mean value and SD of wind speed during each time interval [55] are simulated in Fig 11. To determine the shape parameters of Beta distribution of solar irradiance variations to find the output power from the solar unit, the mean value and standard deviation (SD) of solar irradiance during each segment of time [64] are simulated in Fig 12. Figs 11 and 12 illustrate that the peak of the wind speed and solar irradiance occur at the middle of the day. Using MCS method, in each hour 1000 scenarios are generated to simulate RE variations. Respectively, Figs 13 and 14 show the obtained scenarios of the wind speed and solar irradiance during 12.00–13.00 h by using the MCS technique. Then, these scenarios are shrunk to typical ten scenarios which effectively give an approximation for the original system. Fig 13 illustrates that during the proposed hour the most repeated wind speed values lay between 10 (m/s) and 12 (m/s). Fig 14 illustrates that during the proposed hour the most repeated solar irradiance values lay between 0.7 (Kw/m2) and 0.8 (Kw/m2). The specification of wind turbines and solar unit are proposed in Tables 6 and 7, respectively. The data for 10 thermal units and load demand uncertainty modelling are similar to case 2. The spinning reserve values are 10% of the load. In this case, the thermal units’ ramping capacities are considered and are taken as 75% of the units rated capacity. The commitment schedule of the thermal units is shown in Fig 15. The graphical representation for the generated power from the thermal units is depicted in Fig. The results in Figs 15 and 16 demonstrate that the generation limit constraints, up/down ramping capacity constraints, and minimum up/down time constraints are all meet the requirements proposed previously. The solution of the UC with the consideration of the generation side uncertainty (wind and solar power generation) ensures that the calculated operating cost is more reliable and gives a fairly good simulation for the real power system operation which makes the solution more practical. In comparison with the previous case, it is found that fewer number of units have to be on to cover the load demand at nearly all hours of the day. This indeed reduces the loading on the thermal units except for the first unit which has to work for all day and represents the base generation. As a result, the operating cost reduces with a large rate compared to the cost in the previous case. Despite the increment in the start-up cost, but the fuel cost of thermal units decreases as the wind turbines and solar unit share power with the conventional thermal units to cover the load demand. The cost comparison of this case with the previous case is given in Table 8. The minimum obtained cost by the GTO is 336516.58 ($/day). In other words, the percentage reduction in the operating cost per day with incorporation of RE resources is 19.23%. This means that the amount of savings in the operating cost per day with incorporation of RE resources is 80114.54 ($/day) which equivalent to 29.2418071 ×10^6^ ($/year). The convergence curve for the GTO with incorporation of RE resources is depicted in Fig 17. Fig 18 gives the convergence curves comparison with and without RE resources integration. The convergence rate is fast in the case of without integrated RE resources.

**Table 6:** Specification of wind turbine.

Parameter	Value
Rated output power, *P*_*R*_	40 MW
Rated wind speed, *V*_*R*_	12 m/s
Cut-in-speed, *V*_*in*_	3 m/s
Cut-out-speed, *V*_*out*_	25 m/s

**Table 7:** Specification of solar unit.

Parameter	Value
Solar irradiance for standard conditions, *G*_*std*_	1000 W/m^2^
Cut-in-radiation point, *X*_*c*_	150 W/m^2^

**Table 8:** Costs comparison between case 2 and case 3.

Test case	Start-up cost	Fuel cost	Operating cost
Case 2	3880	412751.12	416631.12
Case 3	5630	330886.58	336516.58

## 6. Conclusion

This work has presented a solution for the single area UC issue. The focus of this work is on a combined power system consists of conventional thermal units, wind turbines, and a solar unit. The goal of the proposed study is to reduce the system operating cost with higher degree of reliability due to the integration of wind and solar energies and the consideration of uncertainty without disturbing the problem constraints. We have applied the GTO that replicates gorilla social behavior to tackle the UC issue. We statistically investigated the impact of integrated RE resources uncertainty and the load uncertainty on solving the UC problem and so the economics of the power system. That has been done by analyzing the results obtained for systems with and without integrated RE resources under deterministic and stochastic models. By the employment of Weibull and Beta PDF, respectively, uncertainties of wind speed and solar irradiance have been simulated. The load demand uncertainty has been simulated using normal PDF. The effectiveness of the GTO is evaluated by considering the deterministic UC problem. The findings of this work suggested that the GTO has superiority in solving the deterministic UC and the incorporation of RE resources with uncertainty consideration has achieved notable cost saving in the operating costs. According to the results derived from the performed studies under three different cases, the following conclusions are deduced: The GTO succeed to achieve better operating cost with fast convergence rate over other algorithms in solving the deterministic UC. The minimum and maximum achieved cost savings per day are 0.2181% and 3.7528%, respectively. The integration of RE resources in the system while considering their uncertainties and fluctuations reduced the load put on the thermal units so the system operation cost decreased by 19.23% per day in comparison with the cost obtained in the second case (Without RE resources integration), and it is found that the system economic performance has been enhanced significantly. The results served as a foundation for additional investigation and advancement in the UC study with various RE resources integration and with uncertainty modelling of various parameters in the system. The main limitation of the proposed solution is the need for energy storage systems to compensate for the instability of the wind and solar energies production. By focusing on this limitation, the findings of the research can be reinforced and the reliability of the system can be enhanced significantly. Addressing these limitations can strengthen the study’s findings. The Future research work focuses on solving the stochastic UC with inclusion the electrical vehicle stations to system and with inclusion multi-types of energy storage systems.

## Abbreviations

Mathematical symbols t: time horizon for a set of *T*
i: thermal power units for a group of *i*
TC: total operating cost
U^t^_i_: operating state of the unit i at time *t*
FC^t^_i_: fuel cost of unit i at time *t*
SC^t^_i_: start-up cost of unit i at time *t*
*a*_*i*_,*b*_*i*_,*c*_*i*_: coefficients of the fuel cost of *i*^*th*^ unit
*P*_*i*_(*t*): generated power from unit *i* at time *t*
${SC}_{i_{hot}}$: hot start-up cost of unit *i*
${SC}_{i_{cold}}$: cold start-up cost of unit *i*
MDT_i_: minimum down time of unit *i*
MUT_i_: minimum up time of unit *i*
$T_{{ON}_{i}}$: time that unit i has been consistently on
$T_{{OFF}_{i}}$: time that unit i has been consistently off
$T_{{cold}_{i}}$: the needed time for unit *i* to become completely cool
*L*_*net*_(*t*): net load at time *t*
P_L_^t^: the system load demand at time *t*
$P_{i_{max}},P_{i_{min}}$: the upper and lower generation limit of unit *i*
$P_{{ramp}_{i}}$: ramp power capacity of unit *i*
SR^t^: the system’s spinning reserve needs at time *t*
*P*_*W*,*out*_(*t*): the wind turbine produced power at time *t*
P_R_: the base produced power from the turbine
V_R_: the base wind speed
V_in_: cut-in speed
V_out_: cut-out speed
V_w_: actual speed of the wind
*P*_*PV*,*out*_(*t*): the solar unit produced power at time *t*
P_sr_: the rated produced power from the solar unit
G_s_: solar irradiance
G_std_: solar irradiance for standard environment conditions
*f*_*V*_^*t*^(*V*): property density function of wind speed at time t
V_t_: wind speed at time t
*k*_*t*_,*c*_*t*_: shape and scale parameter of Weibull distribution at time t
*μ*_*t*_^*V*^, *σ*_*t*_^*V*^: mean value and standard deviation of wind speed at time segment *t*
Γ: gamma function
X_N_: a matrix contains the produced *N* scenarios in 24 hours
*f*_*SR*_^*t*^(*SR*): property density function of solar irradiance
SR_t_: solar irradiance at the *t* th time interval
*α*_*t*_,*β*_*t*_: shape parameters of beta distribution during time segment *t*
*μ*_*t*_^*SR*^, *σ*_*t*_^*SR*^: mean value and standard deviation of solar irradiance at time interval *t*
*PDF*_*L*_(*P*^*t*^_*L*_): property density function of load demand
P^t^_L_: active power of load demand
*μ*^*t*^_*L*_, *σ*^*t*^_*L*_: mean value and standard deviation of the load at time *t*
*GX*(*t*+1): the vector of the candidate solution in the next iteration
*X*(*t*): the vector of current gorilla position
*r*_1_,*r*_2_,*r*_3_,*r*_4_,*r*_5_,*rand*: random vectors between 0 and 1
X_r_: one of the group’s gorillas, picked at random from the entire population
GX_r_: the position vector of one of the gorilla candidates picked randomly
lb: the control variables’ lower limit
ub: the control variables’ upper limit
*C*,*L*,*H*: operators of the artificial gorilla troops optimizer
l: random number varies from -1 to 1
Z: randomized number between −*C* and *C*
*X*_silverback_: position of the silverback gorilla
*GX*_*i*_(*t*): candidate position for each gorilla during iteration *t*
Q: impact force
β: preset parameter
E: the violence impact on the solutions’ dimensions
UC: unit commitment
RE: renewable energy
BRA: backward reduction algorithm
DUC: deterministic unit commitment
SUC: stochastic unit commitment
GA: genetic algorithm
GTO: artificial gorilla troops optimizer
PDFs: probability density functions
MCS: Monte Carlo simulation
SD: standard deviation
